# Bone marrow fibrosis in newly diagnosed multiple myeloma and its correlation with clinicopathological factors

**DOI:** 10.1186/s13000-024-01516-y

**Published:** 2024-07-18

**Authors:** Xiumei Hu, Xiangyang Dai, Xinmeng Guo, Xingran Jiang, Yunlong Li, Hongying Zhao, Jun Lu, Xue Li, Mulan Jin

**Affiliations:** Department of Pathology, Beijing Chao-Yang Hospital, Capital Medical University, 8 Gongren Tiyuchang Nanlu, Chaoyang District, Beijing, 100020 China

**Keywords:** Bone marrow fibrosis, Multiple myeloma, Diagnostic criteria, Pathological characteristics, Infiltration pattern, Prognosis

## Abstract

**Background:**

Bone marrow fibrosis (BMF) severely impacts both the quality of life and the efficacy of diagnostic procedures. However, the correlation between BMF and clinicopathological features, cytogenetic changes, and prognosis of newly diagnosed multiple myeloma (NDMM) remains unclear. This study determined the incidence, patient characteristics, and clinical outcomes of patients with NDMM with BMF.

**Methods:**

The clinical data, histological features, and clinical outcomes of patients with NDMM were collected. Reticular fiber staining was performed on the enrolled cases, and the degree of reticular fiber overgrowth was graded. Patients with MF-2 and MF-3 were classified as the BMF+ group, and those with MF-0 and MF-1 were classified as the BMF- group, and BMF incidence was calculated. The differences in clinical data, histological features, and clinical outcomes between the BMF+ group and the BMF– group were compared.

**Results:**

A consecutive series of 146 patients with NDMM were included. The incidence of MF-0, MF-1, MF-2, and MF-3 was 7.53% (11/146), 34.93% (51/146), 51.37% (75/146), and 6.16% (9/146), respectively. The incidence of BMF—MF-2 and MF-3—was 57.53% (84/146). A significant correlation was identified between the pattern of infiltration and BMF (*P* < 0.001). In the BMF- group, the distribution of cases with interstitial, nodular, and diffuse infiltration of plasma cells was 16 (25.8%), 21 (33.9%), and 25 (40.3%), respectively. Conversely, in the BMF+ group, these values for interstitial, nodular, and diffuse tumor cells were 9 (10.7%), 15 (17.9%), and 60 (71.4%). Furthermore, BMF was associated with a diffuse infiltration pattern. The overall survival (OS) of the BMF+ group (39.1 months; 95% confidence interval [CI]: 34.0–44.3) was lower than that of the BMF- group (45.4 months; 95% CI: 39.5–51.3), but there was no significant difference between the two groups (*P* = 0.221). Univariate and multivariate analyses showed that the BMF+ status was not associated with OS in patients with NDMM (*P* = 0.381 and *P* = 0.748, respectively).

**Conclusions:**

Our findings suggest that BMF is linked to a diffuse infiltration pattern, and its occurrence is not related to the prognosis of patients with NDMM, providing a basis for further exploring the BMF value in NDMM diagnosis and treatment.

**Supplementary Information:**

The online version contains supplementary material available at 10.1186/s13000-024-01516-y.

## Background

Multiple myeloma (MM) is a B-cell malignancy that accounts for 1% of all cancers and approximately 10% of all malignant hematologic neoplasms [1]. Clinically, it is characterized by a pentad of (a) anemia, (b) elevated serum and urine monoclonal paraprotein levels, (c) abnormal bone radiographs and bone pain, (d) hypercalcemia, and (e) renal insufficiency [2]. Recently, proteasome inhibitors (PIs), immunomodulatory drugs (IMiDs), and autologous stem cell transplantation (ASCT) have been widely used to treat patients with MM [3, 4]. Although the application of these treatment methods significantly improves the prognosis of patients with MM, there are significant differences in patient prognosis [5]. Currently, MM remains an incurable disease, and patients eventually die from disease or complications [6]. Widely used MM staging systems currently include the Durie-Salmon (D-S), International Staging System (ISS), and revised ISS (R-ISS). However, owing to the significant heterogeneity of MM, these stages have certain limitations. Exploring the prognostic factors and individualized treatment is particularly important.

Notably, the bone marrow (BM) microenvironment plays an important role in the pathophysiology of MM by supporting the growth and survival of MM cells, inducing their clonal evolution, and suppressing immune cell function [7, 8]. Reticulin is a normal component of the BM that may be increased in BM biopsy samples in a wide variety of neoplastic and non-neoplastic conditions. Bone marrow fibrosis (BMF) refers to the deposition of reticulin or collagen in the BM stromal environment beyond the normal range [9]. Importantly, BMF substantially diminishes the hematopoietic components in the BM, leading to various degrees of cytopenia in patients. This condition often progresses to splenomegaly, weight loss, and other systemic symptoms associated with the disease, severely impacting both the quality of life and the efficacy of diagnostic procedures. However, these clinical manifestations frequently lack distinctiveness and may be overlooked. Therefore, the precise recognition of BMF in patients with NDMM holds significant clinical relevance.

At present, there is still no consensus on the accurate diagnosis and standardized interpretation of BMF in NDMM, making the diagnosis results reported in the previous literature significantly different. It has been previously reported that 8–57% of MM cases are associated with BMF [10–14]. The lack of a clear, defined term for the quantification of BMF has led to the undervaluation of BMF in MM, especially NDMM. Moreover, the correlation between BMF and clinicopathological features, cytogenetic changes, and prognosis of NDMM remains unclear, especially in the era of new drug treatments.

This study aimed to evaluate the prevalence of BMF in patients with NDMM and the relationship between BMF and clinicopathological features, cytogenetic changes, and prognosis to provide a more accurate prognosis for NDMM.

## Methods

### Enrolled cases

The study was approved by the ethical committee of Beijing Chao-Yang Hospital, Capital Medical University. Clinical data of patients with MM in the Department of Pathology of Beijing Chao-Yang Hospital, Capital Medical University, from December 2018 to October 2022 were collected. The inclusion criteria were defined as follows: (a) complete clinical data available; (b) specimens of BM biopsy tissues; and (c) patients diagnosed with NDMM who had not received any prior therapy. Patients with other myeloproliferative neoplasms (MPN) were excluded. Patient data were obtained from our institutional database and through a thorough review of the patients’ electronic medical records. MM diagnosis was established in accordance with the diagnostic criteria set by the International Myeloma Working Group (IMWG) [15]. The clinical features of the patients were collected, including sex, age, M protein type, light-chain type, presence of extramedullary invasion, presence of plasma cell leukemia, D-S stage, ISS stage, and proportion of tumor cells in BM smear. All patients received standard treatment regimens, including PIs, iMiDs monoclonal antibody treatment, and ASCT. Patient prognosis was assessed using medical records and telephone follow-ups until October 30, 2023. Overall survival (OS) was defined as the duration from the date of MM diagnosis to the date of the last follow-up when the patient was alive or dead.

### Hematoxylin and eosin (H&E), immunohistochemical staining (IHC), and pathologic diagnosis

Routine BM pathological evaluation of patients with MM involves H&E and IHC staining. Each patient’s H&E-stained sections underwent morphological review, and diagnoses were independently confirmed by two pathologists, HXM and DXY. Only cases approved by both pathologists were included in this study. Here, we concentrated on examining the following parameters. First, the plasma cell burden was assessed based on the proportion of plasma cells within the BM nucleated cells, categorized as a mild increase (≤ 20%), a moderate increase (21–50%), and a significant increase (> 50%). Second, infiltration patterns, which included interstitial, nodular, and diffuse patterns, were analyzed. The interstitial pattern was characterized by the infiltration of plasma cells into a preserved marrow space, whereas the nodular pattern was defined by aggregates of more than 10 plasma cells within the preserved marrow space. Conversely, the diffuse pattern was identified by the replacement of marrow space with plasma cells, resulting in the loss of fat spaces [16]. Third, plasma cell morphology was assessed. Plasma cells in HE-stained sections were classified into four types: mature, intermediate, immature, and plasmablastic. Mature myeloma cells are characterized by a round, eccentric cartwheel nucleus without nucleoli, abundant basophilic cytoplasm, and a perinuclear hof. Immature myeloma cells have an irregular nucleus with more dispersed chromatin, a higher N/C ratio, and usually prominent nucleoli. Intermediate myeloma cells were excluded, and plasma cell morphology did not meet the criteria for other types. Plasmablastic myeloma cells are large, with increased nuclear polymorphisms and mitotic figures, and resemble diffuse large B-cell lymphoma [17, 18].

IHC staining was performed on formalin-fixed, paraffin-embedded tissues using the ultraView Universal DAB Detection Kit (Ventana Medical Systems, Tucson, AZ, USA) in the BenchMark XT automated immunostainer (Ventana). Proteins CD38 (clone38CO3), CD138 (clone MAB0200), MUM1 (clone MX093), Kappa (clone RAB-0111), Lambda (clone MAB-0357), their reagents, and their primary antibodies were purchased from the Fuzhou Maixin Biotechnologies Development Company (Maixin, Fuzhou, China). All the antibodies used in this study were purchased as ready-to-use working solutions. Immunohistochemical staining methods were performed according to their respective procedures. The repair method used in this study was an ULTRA CC1 alkaline repair solution. The pH was maintained at 8.4 and high-temperature heating was performed at 95–100 °C for the repair. The repair process was performed using the BenchMark XT automated immunostainer. MM was diagnosed when two or more antibodies were positive for CD38, CD138, and Mum-1, and Kappa and Lambda staining showed light-chain restrictive expression, indicating neoplastic plasma cells.

### Reticular fiber staining and result interpretation

Four-micrometer-thick sections were cut from all specimens and stained using a silver impregnation kit for reticulin (Artisan Link Pro, Agilent, Santa Clara, CA, USA; Ventana BenchMark Ultra, Ventana Medical Systems Inc., Oro Valley, AZ, USA). Staining was performed according to the manufacturer’s instructions.

The assessment of BMF followed the European consensus scoring system, which includes four grades (MF-0 to MF-3). The criteria were established as follows: MF-0, indicating scattered linear reticulin without intersections (cross-overs); MF-1, characterized by a loose network of reticulin with numerous intersections, predominantly in perivascular areas; MF-2, defined by a diffuse and dense increase in reticulin with extensive intersections, occasionally presenting only focal bundles of collagen and/or focal osteosclerosis; and MF-3, entailing a diffuse and dense increase in reticulin with extensive intersections and coarse bundles of collagen, often accompanied by significant osteosclerosis.

The evaluation of MF staining was confined to hematopoietic areas [19, 20]. The highest level of fibrotic area exceeding 30% determined the final grade [19, 20]. Assessment of MF was independently confirmed by two pathologists, HXM and DXY, and the observers were blinded to the clinical outcomes. Cases with discrepancies were re-evaluated until a consensus was reached. MF-2 and MF-3 were categorized into the BMF+ group, whereas MF-0 and MF-1 were grouped under the BMF- group.

Masson trichrome staining was employed in cases of MF-3. Masson staining kit was provided by Zhuhai Baso Biotechnology Co., Ltd. The staining solution was improved by Mallory’s three-color staining method. The operation process was performed with reference to the operation guide. With the stain, collagen fibers are stained blue, cytoplasm, muscle fibers, red blood cells, and the nuclei black. This study further graded the results of Masson staining [19]: Grade 0: Perivascular collagen only (normal); Grade 1: Focal paratrabecular and/or central collagen deposition without connecting meshwork; Grade 2: Paratrabecular and/or central deposition of collagen with focally connecting meshwork or generalized paratrabecular apposition of collagen; Grade 3: Diffuse (complete) connecting meshwork of collagen.

### Interphase fluorescence in situ hybridization (iFISH) staining

We collected the patient’s BM fluid, applied heparin anticoagulation, and sorted it using CD138 magnetic beads, followed by iFISH detection.

Mononuclear cells were enriched by the Ficoll-gradient centrifugation method (Ficoll-Paque PLUS; GE Healthcare Bio-Sciences AB, Uppsala, Sweden), and then were assessed using commercially available probes for the regions containing 4p16 (FGFR3)/14q32 (IGH), 11q13 (CCND1)/14q32 (IGH), 14q32 (IGH)/16q32 (MAF), 1q21 and 17p13.1 (TP53) using Vysis IGH/FGFR3 DF FISH Probe Kit, Vysis IGH/CCND1 DF FISH Probe Kit, Vysis IGH/MAF DF FISH Probe Kit, 1q21 CKS1B SpectrumOrange/1p32CDKN2C SpectrumGreen FISH Probe Kit, Vysis TP53/CEP 17 FISH Probe Kit(Vysis/Abbott Molecular, Des Plaines, IL, USA). Slides containing the cells were pretreated, denatured, and hybridized using standard laboratory procedures following the manufacturer’s instructions (Vysis/Abbott Molecular). For each specimen, 200 interphase cells were analyzed [21].

### Statistical analysis

Continuous variables were expressed as medians and ranges, and categorical variables were expressed as absolute frequencies and percentages. IBM SPSS 27.0 (IBM SPSS Inc., Armonk, NY, USA) was used for the statistical analyses. The χ^2^-test with Fisher’s exact tests, as appropriate, was used to examine relationships between categorical variables. Kaplan–Meier survival curves were used to determine the median OS stratified by the presence of BMF. Univariate and multivariate Cox regression analyses were used to determine the association between the clinicopathological characteristics and survival. Statistical significance was set at *P* < 0.05.

## Results

### Clinical and morphological features

The study involved a consecutive series of 146 patients with NDMM. The age of the patients ranged from 29 to 84 years, with an average of 60.84 years and a male:female ratio of 1.44:1. The distribution of the M protein type, light-chain type, D-S stage, ISS stage, and proportion of tumor cells in BM smear in 146 patients with NDMM is shown in Table 1. According to the outcomes of physical examinations and imaging studies, 19 out of 146 patients with NDMM exhibited extramedullary invasion (13.01%), whereas 8 presented with plasma cell leukemia (5.48%). Among 146 patients with NDMM, one case was diagnosed with amyloidosis by lingual mucosa biopsy, and none of the cases were complicated by paraneoplastic syndromes. Overall, 128 patients used a combination of PIs and IMiDs, 2 patients only used IMiDs, 15 patients only used PIs, 22 patients received monoclonal antibody treatment, and 47 patients received ASCT.

**Table 1:** Clinical characteristics of the 146 patients with NDMM in this study

	n (%)
Sex	
Male	87 (59.6%)
Female	59 (40.4%)
Age at diagnosis (years)	
≥ 60	88 (60.3%)
< 60	58 (39.7%)
Pattern of infiltration	
Interstitial	25 (17.1%)
Nodular	36 (24.7%)
Diffuse	85 (58.2%)
Plasma cell burden	
Mild	13 (8.9%)
Moderate	29 (19.9%)
Significant	104 (71.2%)
Plasma cell morphology	
Mature type	73 (50.0%)
Immature type	14 (9.6%)
Intermediate type	58 (39.7%)
Plasmablastic type	1 (0.7%)
M protein type	
IgG type	71 (48.6%)
IgA type	27 (18.5%)
IgD type	5 (3.4%)
Light chain type	41 (28.1%)
Non-secretory type	2 (1.4%)
Light chain type	
Kappa	68 (46.6%)
Lambda	76 (52.0%)
Non-secretory	2 (1.4%)
D-S stage	
I	6 (4.1%)
II	17 (11.6%)
III	123 (84.2%)
ISS stage	
I	26 (17.9%)
II	35 (24.1%)
III	84 (57.9%)
Extramedullary invasion	
With	19 (13.0%)
Without	127 (87.0%)
Plasma cell leukemia	
With	8 (5.5%)
Without	138 (94.5%)
High cytogenetic risk	
With	52 (35.6%)
Without	77 (52.7%)
Missing	17 (11.6%)
Proportion of tumor cells in BM smear	
≤ 10%	9 (6.4%)
> 10%	132 (93.6%)
MF	
MF-0	11 (7.5%)
MF-1	51 (34.9%)
MF-2	75 (51.4%)
MF-3	9 (6.2%)

*Abbreviations*: *BM* Bone Marrow, *D-S* Durie-Salmon, *Ig* Immunoglobulin, *ISS* International Staging System, *MF* Myelofibrosis

**Fig. 1:**
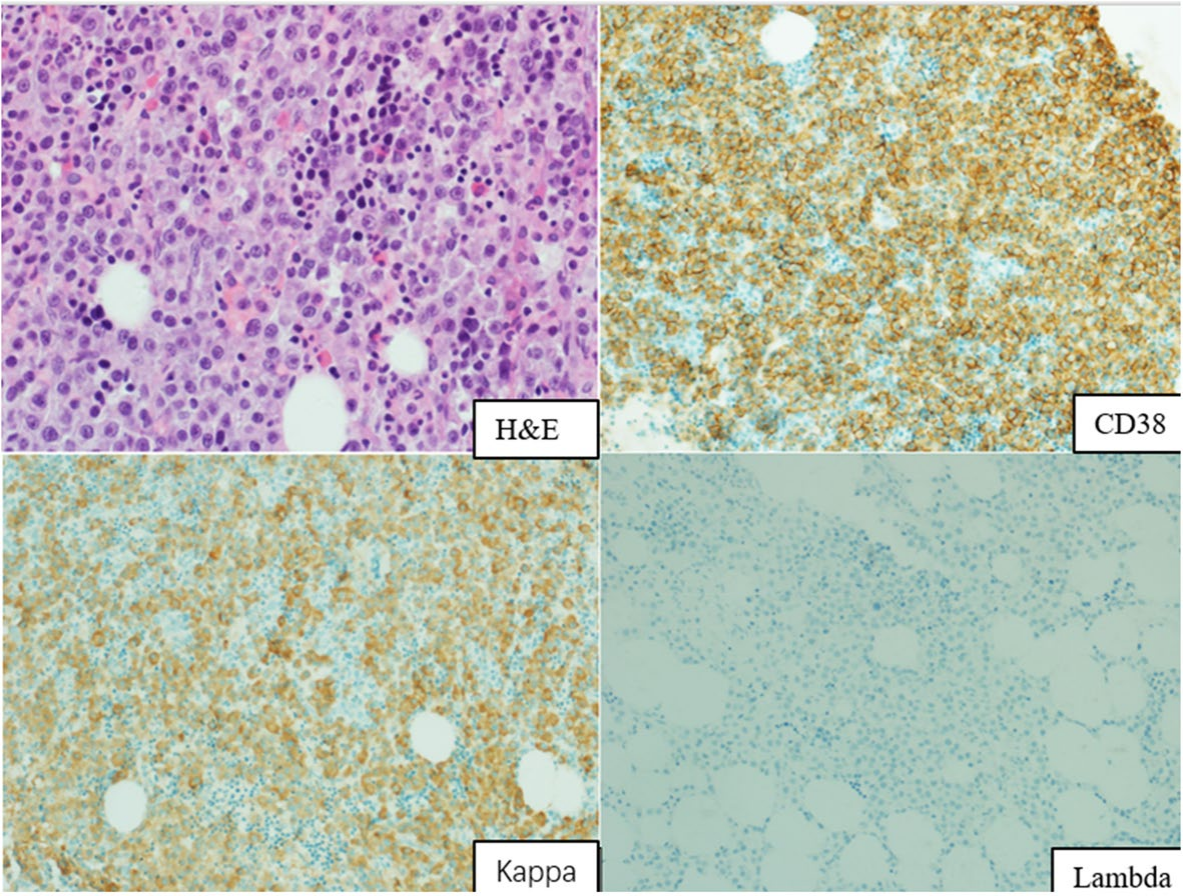
Standard H&E and IHC staining in BM biopsy samples of patients with NDMM. × 200

Morphological parameters, including the morphology of plasma cells, the pattern of infiltration, and the extent of plasma cell burden, were analyzed by BM biopsy. In the 146 NDMM cases, the predominant plasma cell type was mature (73 cases, 50%), and the most common growth pattern was diffuse (85 cases, 58.22%). Notably, a significant increase in plasma cell burden was observed in 104 cases (71.23%). Figure 1 shows the typical pathological morphology and immunophenotype of a patient with NDMM.

**Fig. 2:**
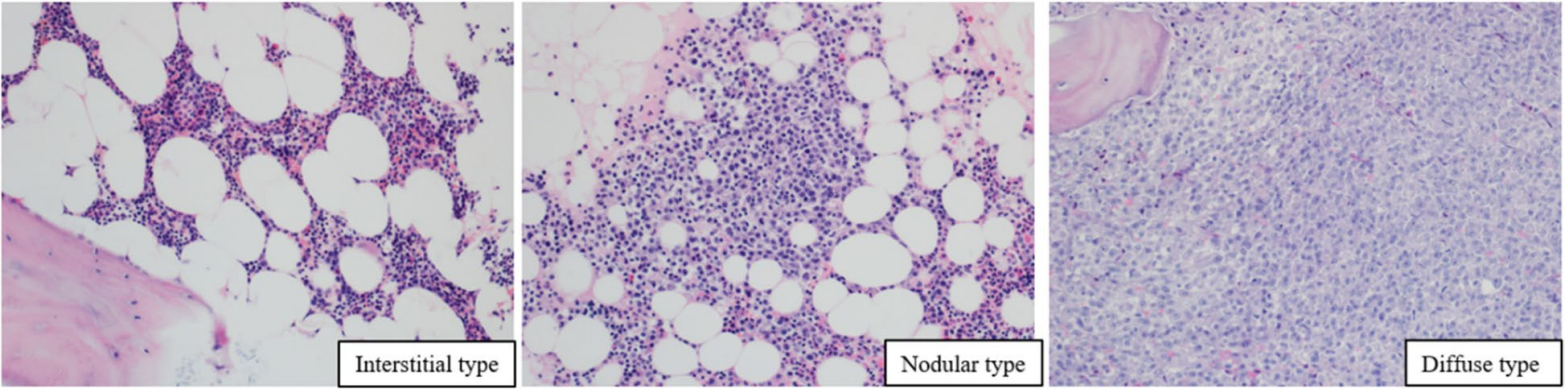
Infiltration patterns of NDMM, including interstitial, nodular, and diffuse types. H&E staining, × 200

Figure 2 shows infiltration patterns of NDMM, including interstitial, nodular, and diffuse types. iFISH detection was performed in 139 patients with NDMM, using probes for 17p13 [del (17p)], 1q21 (1q21 amplification), and translocations including IgH/CCND1 [t (11; 14)], IgH/FGFR3 [t (4; 14)], and IgH/MAF [t (14; 16)]. According to the R-ISS, iFISH detection of 17p13 [del (17p)], IgH/FGFR3 [t (4; 14)], and IgH/MAF [t (14; 16)] is indicative of a high cytogenetic risk. In this study, the incidences of 17p13 [del (17p)], IgH/FGFR3 [t (4; 14)], and IgH/MAF [t (14; 16)] translocations were observed at 18.6% (24/129), 34.1% (44/129), and 23.3% (30/129), respectively. Furthermore, the incidences of 1q21 amplification and IgH/CCND1 translocation were 56.59% (73/129) and 37.98% (49/129), respectively. Notably, the high-risk cytogenetic group represented 59.69% (77/129) of the cases.

**Fig. 3:**
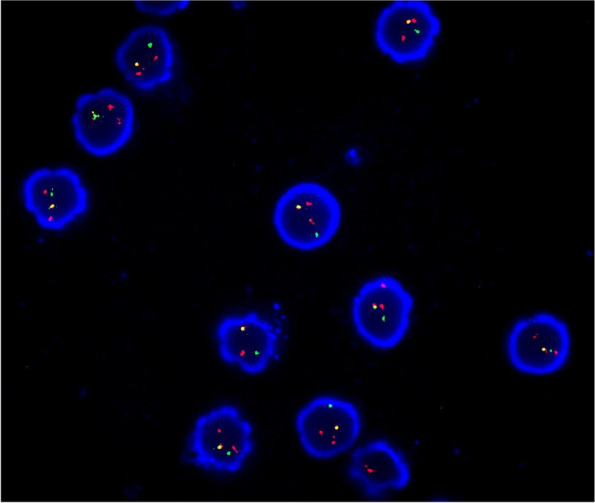
i-FISH detection showing translocations of IgH/FGFR3 [ t (4; 14)]; one yellow signal, one green signal, and one red signal are observed. i-FISH, × 1000

Figure 3 shows characteristic images of iFISH detection of translocations of IgH/FGFR3 [ t (4; 14)].

### Incidence of BMF in NDMM

**Fig. 4:**
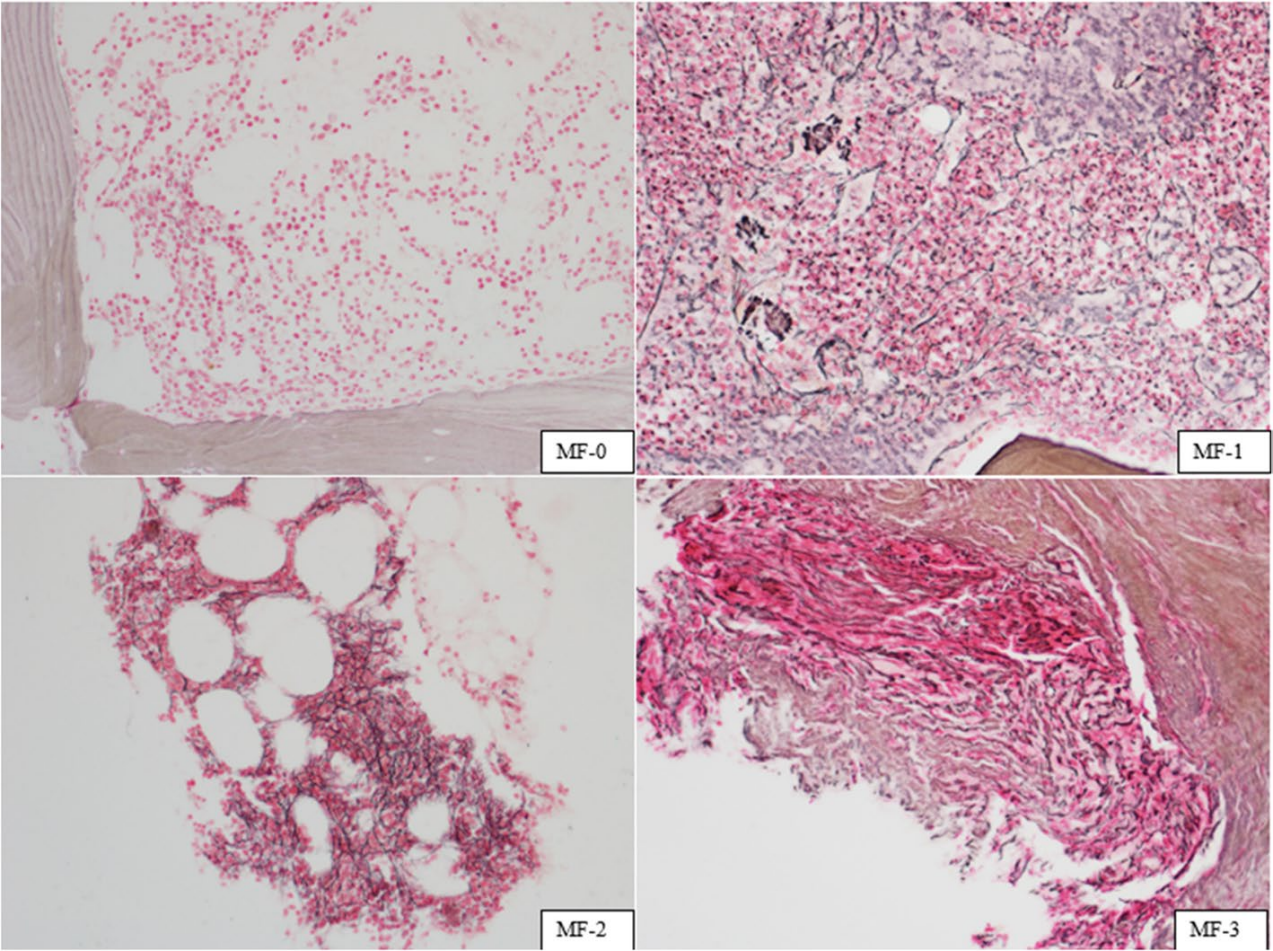
Grading of fiber density and quality, according to the proposed consensus, in BM biopsy specimens of NDMM. Silver impregnation after Gomori, × 200

The incidence of MF-0, MF-1, MF-2, and MF-3 was 7.53% (11/146), 34.93% (51 / 146), 51.37% (75 /146), and 6.16% (9/146), respectively. The incidence of BMF, namely MF-2 and MF-3, was 57.53% (84/146). In the MF-3 group, Masson trichrome staining displayed a green hue, indicative of hyperplastic collagen fibers. The degree of collagen fiber overgrowth in 9 cases of MF-3 NDMM was evaluated. The results were grade 0 in 1 case, grade 1 in 4 cases, grade 2 in 3 cases, and grade 3 in 1 case. No ossification was found in any of the 9 cases. Figure 4 shows characteristic images of the MF-0, MF-1, MF-2, and MF-3 cases.

### Relationship between BMF and clinicopathological features in 146 NDMM

The relationships between BMF and various clinicopathological characteristics are detailed in Table 2. Notably, a significant correlation was observed between the pattern of infiltration and BMF (*P* < 0.001). Specifically, in the BMF- group, the distribution of cases with interstitial, nodular, and diffuse infiltration of plasma cells was 16 (25.8%), 21 (33.9%), and 25 (40.3%), respectively. Conversely, in the BMF+ group, the number of interstitial, nodular, and diffuse tumor cells were 9 (10.7%), 15 (17.9%), and 60 (71.4%), respectively. Regarding plasma cell burden, the BMF+ group exhibited higher percentages than did the BMF- group (78.6% vs. 61.3%); however, this difference was not statistically significant (*P* = 0.069). In patients with ISS III, the percentage in the BMF+ group was higher than that of the BMF- group (65.9% vs. 50.0%), yet this difference was also not statistically significant (*P* = 0.165). Importantly, BMF was not associated with sex, plasma cell morphology, M protein type, light-chain type, D-S stage, extramedullary invasion, plasma cell leukemia, high-risk genetic changes, or the plasma cell ratio in BM smears; the differences were not statistically significant (*P* = 0.483, 0.152, 0.767, 0.232, 0.621, 1.000, 0.537, 0.717, and 0.733, respectively).

**Table 2:** Relationships between BMF and clinicopathological characteristics in 146 patients with NDMM

	BMF– (*n* = 62)	BMF+ (*n* = 84)	*P value*
Pattern of infiltration			
Interstitial	16 (25.8%)	9 (10.7%)	** < 0.001**
Nodular	21 (33.9%)	15 (17.9%)	
Diffuse	25 (40.3%)	60 (71.4%)	
Sex			
Male	39 (62.9%)	48 (57.1%)	0.483
Female	23 (37.1%)	36 (42.9%)	
Plasma cell burden			
Mild increase	8 (12.9%)	5 (6.0%)	0.069
Moderate increase	16 (25.8%)	13 (15.5%)	
Significant increase	38 (61.3%)	66 (78.6%)	
Plasma cell morphology			
Mature type	36 (58.1%)	37 (44.0%)	0.152
Immature type	7 (11.3%)	7 (8.3%)	
Intermediate type	19 (30.6%)	39 (46.4%)	
Plasmablastic type	0 (0.0%)	1 (1.2%)	
M protein type			
IgG type	31 (50%)	40 (47.6%)	0.767
IgA type	12 (19.4%)	15 (17.9%)	
IgD type	3 (4.8%)	2 (2.4%)	
Light chain type	16 (25.8%)	25 (29.8%)	
Non-secretory type	0 (0.0%)	2 (2.4%)	
Light chain type			
Kappa	33 (53.2%)	35 (41.7%)	0.232
Lambda	29 (46.8%)	47 (56.0%)	
Non-secretory	0 (0.0%)	2 (2.4%)	
D-S stage			
I	3 (4.8%)	3 (3.6%)	0.621
II	9 (14.5%)	8(9.5%)	
III	50 (80.6%)	73 (86.9%)	
ISS stage			
I	15 (24.2%)	11 (13.3%)	0.165
II	16 (25.8%)	19 (22.9%)	
III	31 (50.0%)	53 (65.9%)	
Extramedullary invasion			
With	8 (12.9%)	11 (13.1%)	1
Without	54 (87.1%)	73 (86.9%)	
Plasma cell leukemia			
With	3 (4.8%)	5 (6.0%)	0.537
Without	59 (95.2%)	79 (94.0)	
High cytogenetic risk			
With	23 (37.1%)	29 (34.5%)	0.717
Without	31 (50.0%)	46 (54.8%)	
Missing	8 (12.9%)	9 (10.7%)	
Proportion of tumor cells in BM smear			
≤ 10%	3 (5%)	6 (7.4%)	0.733
> 10%	57 (95%)	75 (92.6%)	

*Abbreviations*: *BMF-* Bone Marrow Fibrosis negative, *BMF*+ Bone Marrow Fibrosis positive, *D-S* Durie-Salmon, *Ig* Immunoglobulin, *ISS* International Staging System

### Relationship between BMF and OS

**Fig. 5:**
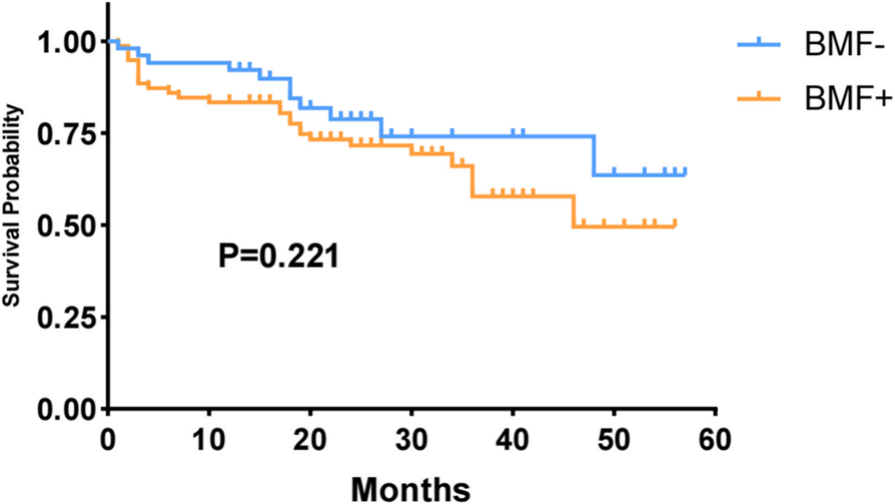
Kaplan–Meier cumulative overall survival (OS) analysis according to the presence or absence of BMF

In total, 129 patients were successfully followed up until October 30, 2023. The mean follow-up interval was 20.0 months (range, 1–57 months). The OS of the BMF+ group (39.1 months; 95% CI: 34.0–44.3) was lower than that of the BMF– group (45.4 months; 95% CI: 39.5–51.3), but there was no significant difference between the two groups (*P* = 0.221). Figure 5 shows Kaplan–Meier cumulative overall survival (OS) analysis according to the presence or absence of BMF.

Moreover, univariate analysis of the OS revealed that the pattern of infiltration, plasma cell morphology, and ISS were associated with poor survival in patients with NDMM (*P* = 0.004, 0.039, and 0.04, respectively). The final multivariate analysis identified the infiltration pattern as having prognostic importance (*P* = 0.029). However, the BMF+ status was not associated with OS in patients with NDMM (*P* = 0.381 and *P* = 0.748, respectively) (Table 3).

**Table 3:** Univariate and multivariate analysis of OS in 146 patients with NDMM

	Univariate			Multivariate		
	HR	95% CI	*P*	HR	95%CI	*P*
Pattern of infiltration			**0.004**			**0.03**
Interstitial	2.937	1.526–1.655	**0.001**	2.065	1.027–4.152	0.42
Nodular	1.076	0.531–2.183	0.839	0.726	0.339–1.554	0.41
Diffuse (ref.)	–	–	–	–	–	–
Plasma cell morphology			**0.039**			0.07
Mature type	0.693	0.293–1.637	0.403	0.952	0.396–2.289	0.91
Immature type	0.986	0.422–2.303	0.973	1.027	0.435–2.421	0.95
Intermediate type	13.225	1.489–117.482	**0.021**	17.87	1.847–172.909	**0.01**
Plasmablastic type	–	–	–	–	–	–
ISS stage			**0.04**			0.13
1	0.224	0.069–0.723	**0.012**	0.261	0.077–0.882	**0.03**
2	0.521	0.244–1.112	0.092	0.599	0.275–1.306	0.2
3	–	–	–	–	–	–
MF (0/1 VS 2–3)	0.78	0.448–1.359	0.381	1.101	0.611–1.984	0.75
Sex	0.868	0.498–1.511	0.616			
Age at diagnosis	1.484	0.825–2.669	0.188			
Plasma cell burden			0.18			
Mild increase	2.047	0.946–4.429	0.069			
Moderate increase	1.301	0.636–2.660	0.471			
Significant increase	–	–	–			
M protein type			0.199			
IgG type	0.469	0.063–3.519	0.462			
IgA type	0.284	0.034–2.355	0.244			
IgD type	1.402	0.145–13.572	0.771			
Light chain type	0.541	0.071–4.113	0.553			
Non-secretory type	–	–	–			
Light chain type			0.768			
Kappa	0.483	0.065–3.598	0.477			
Lambda	0.474	0.063–3.559	0.468			
Non-secretory	–	–	–			
DS stage			0.622			
1	0	0–4.233e + 254	0.968			
2	0.661	0.287–1.520	0.33			
3	–	–	–			
Extramedullary invasion	0.798	0.360–1.769	0.578			
Plasma cell leukemia	0.486	0.192–1.227	0.127			
High cytogenetic risk	0.689	0.362–1.311	0.256			
Proportion of tumor cells in BM smear	1.232	0.439–3.457	0.692			

*Abbreviations*: *BMF-* Bone Marrow Fibrosis negative, *BMF*+ Bone Marrow Fibrosis positive, *D-S* Durie-Salmon, *Ig* Immunoglobulin, *ISS* International Staging System, *CI* Confidence interval

## Discussion

This study possesses distinct characteristics. 1) Case selection: we exclusively included cases of NDMM to mitigate potential influences of treatment and other factors on reticular fiber overgrowth. 2) Assessment of reticular fiber overgrowth (RFO): RFH in NDMM was evaluated using reticular fiber staining. Krzyzaniak et al. [12] initially classified myelofibrosis in 297 MM cases. Their findings indicated that BMF was suspected in H&E-stained sections in 48 (28.28%) of these cases and confirmed by reticulin fiber staining in 26 (8.8%) cases, which is lower than our observed rate. This discrepancy is primarily attributed to instances where RFH was inconspicuous in HE-stained sections and was not subsequently verified by reticular fiber staining. 3) RFH evaluation: the extent of RFH was assessed solely in hematopoietic areas, with the final grade determined by the most severe fibrosis area exceeding 30% [19, 20]. However, previous studies have not reported on this topic. Moreover, the grading of BMF remains an ongoing discussion. Currently, there are no unified diagnostic criteria for BMF in NDMM, resulting in variability in the reported incidence in prior literature [10–14]. Recently, Paul et al. [22] evaluated the BMF of 253 patients with NDMM. The results showed that 122 (48.2%) patients had detectable BMF, including MF-1, MF-2, and MF-3, while 131 (51.8%) had no BMF, namely MF-0. This finding is somewhat different from the results of our study. We suspect the reasons are as follows: First, fiber density was assessed only in hematopoietic areas, and the final score was determined by the highest-grade present in at least 30% of the marrow area in our research. Second, in our study, patients with stages I, II and III accounted for 17.9%, 24.1%, and 57.9%, respectively, while in Paul et al. ‘s study, patients with stages I, II and III accounted for 31.2%, 30.6%, and 38.2%, respectively. The proportion of stage III patients in our study was significantly higher than that of stage I patients. Third, a difference existed between the observers, especially in the case of MF-1. Previous studies reported high inter-observer differences, with a kappa value of only 0.373 (95% CI 0.174–0.573) [19].

Currently, the semi-quantitative grading of RFH, categorized into MF-0, MF-1, MF-2, or MF-3 based on the extent of overgrowth, is widely acknowledged. A significant debate centers on whether the MF-1 group should be classified as BMF. The diagnostic criterion for primary BMF requires an MF grade of 2 or higher [20]. In our clinical practice, reticular fiber staining is routinely conducted on all BM biopsy specimens, and a mild increase in reticular fibers (MF-1) is frequently observed in non-neoplastic lesions. In our study, statistical analysis was conducted on 11 patients in the MF-0 group and 51 patients in the MF-1 group, revealing no statistically significant differences between the MF-0 and the MF-1 groups (data was shown in Supplement Table 1). Consequently, in our research, a grade of MF ≥ 2 was identified as indicative of BMF, leading to the categorization of MF-2 and MF-3 into the BMF+ group, whereas MF-0 and MF-1 were classified under the BMF- group.

This study further investigated the association between clinicopathological features and BMF in patients with NDMM. The results revealed a connection between BMF and a diffuse infiltration pattern, corroborating the findings of Bartl et al. [10] Notably, since reticulin fibrosis is often reversible, earlier studies have documented that the presence of tumor cells in BM diminishes following MM treatment, with a reduction in fibrosis observed in 71% of cases [23]. This also supports the argument that BMF is related to the infiltration of tumor cells. Furthermore, our study revealed that characteristics indicative of a high tumor burden, such as a notable increase in plasma cell load and advanced D-S stage, were significantly more prevalent in the BMF+ group than in the BMF– group. These factors are recognized prognostic indicators for survival in MM. In a related study, Subramanian et al. examined 44 patients with MM and found that 9 cases (20.5%) exhibited BMF, predominantly of the plasmablastic type (8 cases), a proportion significantly higher than that of other types [14]. Contrastingly, our study did not reach a similar conclusion. This was potentially attributable to the exclusive inclusion of patients with NDMM and the minimal incidence of the plasmablastic type (merely one case).

Combined extramedullary invasion or plasma cell leukemia are poor prognostic factors for NDMM. Koshiishi et al. studied 91 cases of NDMM and found that 5.49% (5/91) had extramedullary disease, and the patients with BMF tended to exhibit extramedullary disease [23]. In our study, 13.0% (19/146) of the patients with MM presented with extramedullary invasion, and 5.5% (8/146) were diagnosed with plasma cell leukemia. This discrepancy was not observed in our study, and two potential reasons may account for this: 1) the incidence of extramedullary disease in our study was 13.0%, which is higher than that reported in the study by Koshiishi et al., and 2) in our study, a diagnosis of BMF was made with an MF ≥ 2, whereas Koshiishi et al. classified BMF at a threshold of MF ≥ 1.

The relationship between BMF and the prognosis of patients with MM remains unclear. Previous case series studies have indicated that patients with MM and BMF have poorer survival [14, 24, 25]. Sailer et al. stated that patients with BMF had a survival time of only 18 months [26]. Barry Paul et al. conducted reticular fiber staining in 393 patients with NDMM and conducted a follow-up, with a median duration of 83 months (range: 3.9–212 months). The findings revealed that the presence of BMF correlated with reduced OS and PFS [22]. However, our study did not provide conclusive evidence. The ISS staging, in conjunction with β2-MG and albumin levels, was employed to assess the patient’s prognosis. Our study found no marked differences between BMF and ISS staging. Our results indicated that patients with BMF had a shorter survival than those without BMF, albeit the differences were not statistically significant. Moreover, further univariate and multivariate survival analyses revealed that MF did not impact OS. A probable explanation is that the patients in our study received a standardized regimen comprising IMiD, PIs, and ASCT, contributing to their prolonged survival in comparison to the previously mentioned studies (45.4 months in the BMF– group, 39.2 months in the BMF+ group). Megumi Koshiishi [23] and colleagues analyzed 91 cases of NDMM treated with PIs and IMiDs. Their findings indicated that BMF did not significantly affect treatment response, OS, or PFS. Therefore, it can be inferred that the occurrence of BMF may not influence the prognosis of patients receiving modern treatment. Besides MM, BMF characteristics associated with other neoplastic diseases have been reported. An increase in reticulin fibers in BM has been observed in various hematological malignancies, including MPN, myelodysplastic syndrome (MDS), and acute myeloid leukemia [27, 28]. Notably, in chronic myelogenous leukemia (CML), myelofibrosis has been identified as a significant predictor of therapeutic efficacy and patient outcomes [29]. In MDS, patients with BMF exhibit significantly worse survival compared with patients with no BMF [30].

The significance of reticular fiber staining in diagnosing parathyroid tumors, adrenocortical adenomas, and pituitary adenomas is well-established [31, 32]. Reticular fibers, challenging to discern under H&E staining, become readily observable post-immersion in a silver ammonia solution and subsequent reduction with formaldehyde, yielding a black color. This method simplifies the examination of the characteristics of reticular fibers using an optical microscope. Characterized by its speed, cost-effectiveness, stability, and straightforward interpretation, reticular fiber staining effectively reveals variations in quantity. All specimens in this study were decalcified using a strongly acidic solution. The decalcified specimens were effectively stained with reticular fibers, demonstrating consistency and underscoring the maturity and stability of this widely used technique in clinical diagnosis.

The BM microenvironment is complex and comprises extracellular matrix proteins, cytokines, bone marrow stromal cells, mesenchymal stem cells, osteoblasts, osteoclasts, inflammatory cells, megakaryocytes, and microvessels [33]. Notably, substantial evidence now supports the notion that crosstalk and interactions among these components are crucial for myeloma cell proliferation and disease progression [25]. Plasma cells and BM stromal cells are known to secrete high levels of transforming growth factor-β1, and megakaryocytes produce cytokines that mediate tumor plasma cell proliferation. These factors can stimulate myofibroblasts to synthesize collagen, promote the synthesis of BM extracellular matrix proteins, and inhibit the degradation of extracellular matrix components, eventually leading to the occurrence of BMF [34, 35].

This study has some limitations. First, this study was a retrospective study, and the clinical follow-up time was not long enough, so there are inevitably some unnoticed bias factors and confounding factors. Second, the inherent heterogeneity of myeloma may have impacted the results. Third, sampling variability was a concern due to the focal distribution of the disease. Fourth, the specimens were treated with strong acid decalcification, precluding further next-generation sequencing and FISH detection. Finally, the treatment regimens varied among patients, potentially influencing prognosis outcomes. Therefore, future studies with larger samples and rigorous methodologies are essential to provide more comprehensive information and reveal prognostic effects.

## Conclusions

In conclusion, achieving an accurate diagnosis and ensuring a standardized interpretation of the BMF state in patients with NDMM is of utmost importance. This study investigated a cohort of patients with comprehensive data, encompassing laboratory tests, imaging studies, histological examination, iFISH analysis, and prognostic outcomes, to further elucidate the significance of BMF in NDMM. BMF was recognized to be associated with a diffuse infiltration pattern; importantly, no correlation was found between the presence of BMF and the prognosis of individuals with NDMM. Our study provides a basis for further exploring the value of BMF in the diagnosis and treatment of NDMM.

### Supplementary Information

Supplementary Material 1.

## Data Availability

No datasets were generated or analysed during the current study.

## Abbreviations

BM: Bone Marrow
BMF: Bone Marrow Fibrosis
D-S: Durie-Salmon (referring to the Durie-Salmon staging system)
H&E: Hematoxylin and Eosin
Ig: Immunoglobulin
iFISH: Interphase Fluorescence in Situ Hybridization
IMWG: International Myeloma Working Group
IHC: Immunohistochemistry
ISS: International Staging System
MF: Myelofibrosis (in the context of grading bone marrow fibrosis)
MM: Multiple Myeloma
NDMM: Newly Diagnosed Multiple Myeloma
OS: Overall Survival
R-ISS: Revised International Staging System
RFO: Reticular Fiber Overgrowth
TGF-β: Transforming Growth Factor Beta
MDS: Myelodysplastic Syndrome
MPN: Myeloproliferative Neoplasms
CML: Chronic Myelogenous Leukemia
PIs: Proteasome Inhibitors
IMiDs: Immunomodulatory Drugs
ASCT: Autologous Stem Cell Transplantation
