# Supplementary material for: Bone marrow fibrosis in newly diagnosed multiple myeloma and its correlation with clinicopathological factors
Source: Diagn Pathol. 2024 Jul 18;19:99. doi: 10.1186/s13000-024-01516-y (PMC11256401; doi:10.1186/s13000-024-01516-y)
Supplement: Supplementary file 1 — Supplementary Material 1. [file 13000_2024_1516_MOESM1_ESM.docx]

**The relationship between clinicopathological features of NDMM patients in MF-0 group and MF-1 group.**

|  |  | MF–0 (n=11) | MF-1 (n=51) | *p value* |
| --- | --- | --- | --- | --- |
| Pattern of infiltration | Interstitial | 4（36.4%） | 12（23.5%） | 0.703 |
|  | Nodular | 3（27.3%） | 18（35.3%） |  |
|  | Diffuse | 4（36.4%） | 21（41.2%） |  |
| Sex | Male | 8（72.7%） | 31（60.8%） | 0.516 |
|  | Female | 3（27.3%） | 20（39.2%） |  |
| plasma cell burden | Mild increase | 0（0.0%） | 8（15.7%） | 0.549 |
|  | Moderate increase | 3（27.3%） | 13（25.5%） |  |
|  | Significant increase | 8（72.7%） | 30（58.5%） |  |
| plasma cell morphology | Mature type | 5(45.5%) | 31（60.8%） | 0.572 |
|  | Immature type | 5(45.5%) | 14(27.5%) |  |
|  | Intermediate type | 1（9.1%） | 6(11.8%) |  |
|  | Plasmablastic type | 0（0.%） | 0(0.0%) |  |
| M protein type | IgG type | 4（36.4%） | 27（52.9%） | 0.132 |
|  | IgA type | 3（27.3%） | 9（17.6%） |  |
|  | IgD type | 2（18.2%） | 1（2.0%） |  |
|  | Light chain type | 2（18.2%） | 14（27.5%） |  |
|  | Non-secretory type | 0（0.0%） | 0（0.0%） |  |
| Light chain type | Kappa | 6（54.5%） | 27（52.9%） | 1.000 |
|  | Lambda | 5（45.5%） | 24（47.1%） |  |
|  | Non-secretory | 0（0.0%） | 0（0.0%） |  |
| D-S stage | Ⅰ | 0（0.0%） | 3（5.9%） | 0.305 |
|  | Ⅱ | 0（0.0%） | 9(17.6%) |  |
|  | Ⅲ | 11（100.0%） | 39（76.5%） |  |
| ISS stage | Ⅰ | 0（0.0%） | 15（29.4%） | 0.100 |
|  | Ⅱ | 3（27.3%） | 13（25.5%） |  |
|  | Ⅲ | 8（72.7%） | 23（45.1%） |  |
| Extramedullary invasion | With | 0（0.00%） | 8（15.7%） | 0.330 |
|  | Without | 11（100.0%） | 43（84.3%） |  |
| Plasma cell leukemia | With | 0（0.0%） | 3（5.9%） | 1.000 |
|  | Without | 11（100.0%） | 48（94.1） |  |
| High cytogenetic risk | With | 5 (45.5%) | 26(51.0%) | 0.943 |
|  | Without | 3 (27.3%) | 20 (39.2%) |  |
|  | Missing | 3 (27.3%) | 5 (9.8%) |  |
| Proportion of tumor cells in BM smear | ≤10% | 0(0.0%) | 3 (5.9%) | 1.000 |
|  | >10% | 9 (100%) | 748(94.1%) |  |

Abbreviations: BM: Bone Marrow；BMF: Bone Marrow Fibrosis; D-S: Durie-Salmon; Ig: Immunoglobulin; ISS: International Staging System.
